# ABCA7 Regulates Brain Fatty Acid Metabolism During LPS-Induced Acute Inflammation

**DOI:** 10.3389/fnins.2021.647974

**Published:** 2021-04-07

**Authors:** Tomonori Aikawa, Yingxue Ren, Marie-Louise Holm, Yan W. Asmann, Amer Alam, Michael L. Fitzgerald, Guojun Bu, Takahisa Kanekiyo

**Affiliations:** ^1^Department of Neuroscience, Mayo Clinic, Jacksonville, FL, United States; ^2^Department of Health Sciences Research, Mayo Clinic, Jacksonville, FL, United States; ^3^The Hormel Institute, University of Minnesota, Austin, MN, United States; ^4^Lipid Metabolism Unit, Center for Computational and Integrative Biology, Massachusetts General Hospital, Harvard Medical School, Boston, MA, United States

**Keywords:** Alzheimer’s disease, fatty acids, inflammation, lipopolysaccharide, metabolomics, ABCA7

## Abstract

The ATP binding cassette subfamily A member 7 (*ABCA7*) gene is one of the significant susceptibility loci for Alzheimer’s disease (AD). Furthermore, ABCA7 loss of function variants resulting from premature termination codon in the gene are associated with increased risk for AD. ABCA7 belongs to the ABC transporter family, which mediates the transport of diverse metabolites across the cell membrane. ABCA7 is also involved in modulating immune responses. Because the immune system and lipid metabolism causatively engage in the pathogenesis of AD, we investigated how ABCA7 haplodeficiency modulates the metabolic profile in mouse brains during acute immune response using a metabolomics approach through LC/Q-TOF-MS. Peripheral lipopolysaccharide (LPS) stimulation substantially influenced the metabolite content in the cortex, however, the effect on metabolic profiles in *Abca7* heterozygous knockout mice (*Abca7*^±^) was modest compared to that in the control wild-type mice. Weighted gene co-expression network analysis (WGCNA) of the metabolomics dataset identified two modules influenced by LPS administration and ABCA7 haplodeficiency, in which glycerophospholipid metabolism, linoleic acid metabolism, and α-linolenic acid metabolism were identified as major pathways. Consistent with these findings, we also found that LPS stimulation increased the brain levels of eicosapentaenoic acid, oleic acid, and palmitic acid in *Abca7*^±^ mice, but not control mice. Together, our results indicate that ABCA7 is involved in the crosstalk between fatty acid metabolism and inflammation in the brain, and disturbances in these pathways may contribute to the risk for AD.

## Introduction

ATP-binding cassette (ABC) transporters regulate the transport of a variety of substances across membranes as the importers, exporters, and extruders (Rees et al., 2009; Thomas and Tampe, 2018), thereby mediating various critical pathways to maintain systemic homeostasis (Thomas and Tampe, 2020). In particular, the subfamilies of ABCA, ABCB, ABCC, ABCD, and ABCG are predominantly involved in the transport of lipids including cholesterol, bile acids, phospholipids, and sphingolipids (Tarling et al., 2013; Neumann et al., 2017). While those transporters are highly expressed in the brain and mediate lipid metabolism, their substantial contributions to inflammatory processes have been also implicated in neurodegenerative diseases (Kim et al., 2008; Kooij et al., 2012). Since lipid and lipoprotein metabolism in microglia is affected in pathological conditions such as Alzheimer’s disease (AD) and multiple sclerosis (Loving and Bruce, 2020), the significance of defining crosstalk between lipid metabolism and the immune system in the brain has been increasingly made apparent.

Of note, accumulating genetic evidence collected through genome-wide studies (GWASs) has confirmed *ABCA7* variants to be late-onset AD susceptibility loci, which include rs3764650, rs3752246, and rs115550680 (Hollingworth et al., 2011; Naj et al., 2011; Guerreiro et al., 2013; Jonsson et al., 2013). Subsequent whole genome sequencing studies have shown that carrying one allele of *ABCA7* with a premature termination codon mutation that results in loss of function significantly increases the risk for AD (Cuyvers et al., 2015; Steinberg et al., 2015). ABCA7 and ABCA1 are the closest homologs, sharing 54% sequence identity (Kaminski et al., 2000). ABCA7 also shares functional attributes with ABCA1, such as mediating the efflux of cellular lipids including cholesterol and phospholipids (Wang et al., 2000, 2003; Fitzgerald et al., 2002; Abe-Dohmae et al., 2004; Tomioka et al., 2017). Indeed, lipid metabolism is substantially involved in AD pathogenesis through multiple pathways including amyloid precursor protein (APP) processing, immune responses, and energy balance (Chew et al., 2020). ABCA7 has been also shown to regulate phagocytosis in immune cells (Abe-Dohmae and Yokoyama, 2020). Furthermore, our previous work found that mRNA levels of inflammatory cytokines in the brain during acute immune responses induced by lipopolysaccharide (LPS) stimulation are compromised in both homozygous and heterozygous ABCA7 knockout mice compared to control mice (Aikawa et al., 2019). Therefore, to further investigate the crosstalk between inflammation and lipid metabolism through ABCA7, this study used a metabolomics approach to explore how ABCA7 haplodeficiency influences the LPS-induced changes in metabolite profiles in mouse brains. Our findings demonstrate that ABCA7 contributes to the metabolism of brain fatty acids during LPS-induced acute inflammation.

## Materials and Methods

### Animals

All animal experiments were approved by the Mayo Clinic Institutional Animal Care and Use Committee (IACUC) and were in accordance with the National Institutes of Health Guide for the Care and Use of Laboratory Animals. *Abca7* knockout mice (*Abca7^–/–^*) (Kim et al., 2005) were crossbred with wild-type C57BL/6 inbred mice. Littermate male *Abca7*^+^*^/^*^+^ (control) and *Abca7* heterozygous knockout mice (*Abca7*^±^) mice were intraperitoneally injected with LPS (5 mg/kg; *Escherichia coli* O26:B6; Sigma, L2654) or vehicle at the age of 2–3 months, and used for the experiments 3.5 h after the injection.

### Qualitative Large-Scale Profiling for Metabolomics

The non-targeted metabolomics and non-esterified fatty acid analysis were conducted by the Mayo Clinic Metabolomics Core. Tissue homogenates were deproteinized with six times the volume of cold acetonitrile:methanol (1:1 ratio), kept on ice with intermittent vortexing for 30 min at 4°C, then centrifuged at 18,000 × *g*. 13C6-phenylalanine (3 μl at 250 ng/μl) was added as an internal standard to each sample before deproteinization. The supernatants were divided into two aliquots and dried down for analysis on a Quadrupole Time-of-Flight Mass Spectrometer (Agilent Technologies 6550 Q-TOF) coupled with an Ultra High-Pressure Liquid Chromatograph (1290 Infinity UHPLC Agilent Technologies). Profiling data were acquired under both positive and negative electrospray ionization conditions over a mass range of 100–1,200 m/z at a resolution of 10,000–35,000 (separate runs). Metabolite separation was achieved using two columns of differing polarity, a hydrophilic interaction column (HILIC, ethylene-bridged hybrid 2.1 mm × 150 mm, 1.7 mm; Waters) and a reversed-phase C18 column (high-strength silica 2.1 mm × 150 mm, 1.8 mm; Waters). For each column, the run time was 20 min using a flow rate of 400 μl/min. A total of four runs per sample were performed to give maximum coverage of metabolites. Samples were injected in duplicates, and a quality control sample made up of a subset of samples from the study will be injected several times during a run. All raw data files obtained were converted to compound exchange file format using Masshunter DA reprocessor software (Agilent). Mass Profiler Professional (Agilent) was used for data alignment and to convert each metabolite feature (m/z × intensity × time) into a matrix of detected peaks for compound identification. Each component was assigned a putative identification (ID) through the Metlin database or a mass (m/z) value. Mass accuracy of the Q-TOF method was <5 ppm with retention time precision better than 0.2%. Fold changes as >1.2 or <−1.2 were detected with a precision of 4%. Amounts of non-esterified fatty acids were measured against a standard curve on the Thermo TSQ Quantum Ultra mass spectrometer (West Palm Beach, FL, United States) coupled with a Waters Acquity UPLC system (Milford, MA, United States).

### Weighted Gene Co-expression Network Analysis (WGCNA)

We performed WGCNA of the metabolomics dataset through R package (Langfelder and Horvath, 2008). To identify metabolites that are correlated with the 4 sample groups, we used a power of 5, a minimum module size of 40 metabolites, and a minimum height for merging modules at 0.25 to build an unsigned network. To assess the correlation of modules to the mouse groups, we defined control mice administrated with LPS as 1, *Abca7*^±^ mice administrated with LPS as 2, control mice without LPS administration as 3, and *Abca7*^±^ mice without LPS administration as 4. Metabolites with high connectivity in their respective modules were considered hub metabolites. Intramodular metabolite-metabolite connections were visualized using VisANT. Pathway analysis was conducted using Kyoto Encyclopedia of Genes and Genomes (KEGG) in Metaboanalyst 4.0.

### Statistics

In the metabolomics profiling, potential metabolic signatures with a false discovery rate (FDR) < 0.05 were identified. Statistical significance for the effects of LPS administration on individual metabolite was determined by Tukey *post hoc* analysis after analysis of variance (ANOVA). For the comparison of non-esterified fatty acid levels, statistical significance for the effects of LPS administration each fatty acid was determined by unpaired student-*t* test. A *p*-value less than 0.05 was considered as statistically significant.

## Results

### ABCA7 Haplodeficiency Impacts the Metabolomic Changes Induced by LPS Administration in Mouse Brains

**FIGURE 1:**
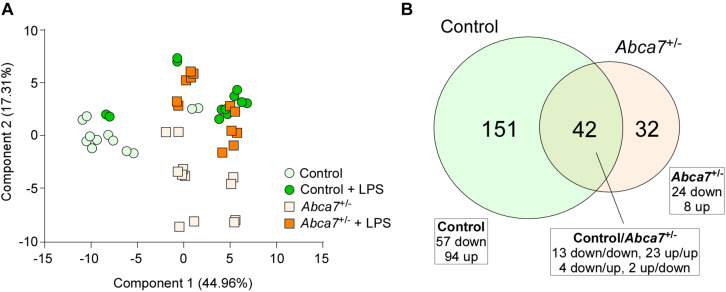
Influence of ABCA7 haplodeficiency on LPS-induced metabolomic changes in mouse brains. Metabolomics was conducted in the cortex of control and *Abca7*^±^ mice (*N* = 6/each) administrated with or without LPS. **(A)** Principal component analysis (PCA) of metabolites from control and *Abca7*^±^ mice with or without LPS administration. **(B)** Venn diagram of the metabolites differently induced by LPS administration in control and/or *Abca7*^±^ mice.

Non-targeted metabolomics was performed on the cortical samples from control and *Abca7*^±^ mice administrated with or without intraperitoneal LPS injection. While a total of 5,593 metabolites were detected, principal component analysis (PCA) demonstrated the distinct clustering among the four groups (Figure 1A). The PCA plot showed the separation of samples with 44.96% variance captured by PC1 and 17.31% variance captured by PC2. In addition, 253 metabolites with FDR < 0.05 were identified in the 4 groups of mice (Supplementary Data). Among them, we identified 193 and 74 metabolites differentially induced by LPS administration in control and *Abca7*^±^ mice (raw *p*-value < 0.05), respectively, with an overlap of 42 metabolites (Figure 1B and Table 1). While 119 metabolites were increased by LPS administration in control mice, 74 metabolites were decreased. In *Abca7*^±^ mice, LPS administration induced the upregulation of 35 metabolites and the downregulation of 39 metabolites. Thus, these results indicate that peripheral LPS injection modulates the metabolome in mouse brains, in which ABCA7 haplodeficiency diminishes the changes in metabolic profiles.

**TABLE 1:** Metabolites affected by LPS administration in the mouse brains.

Compound	Control		*Abca7*^±^		Ionization mode	MS1 composite spectrum (m/z)
		
	FC	*p*-value	FC	*p*-value		
C24 H9 N11 O4	−1229.987	1.74E–04	−556.729	2.24E–04	HILIC+	(516.0915, 2651.58) (517.0916, 692.18)
Quinacetol	703.429	1.30E–03	144.123	1.90E–02	HILIC+	(188.0709, 1615.56) (205.0975, 1986.9) (206.1013, 176.11)
C21 H43 N4 O4	138.129	9.79E–03	687.350	5.39E–04	HILIC+	(416.3347, 4091.88) (417.3392, 1173.53) (433.3659, 204.09)
His Ala	2.108	8.07E–04	1.985	2.00E–03	HILIC+	(249.0964, 555.13) (227.1139, 44894.72) (228.1171, 4758.09) (229.119, 466.04)
Carnosine	2.012	1.70E–04	1.753	2.79E–04	HILIC−	(225.0992, 13183.61) (226.1022, 1362.8)
PE [15:1(9Z)/22:0]	−4.176	2.91E–03	−2.244	N.S.	C18+	(760.5844, 113759.48) (761.5878, 50487.69)
POV-PC Esi + 12.015995	−3.239	1.15E–03	−1.130	N.S.	C18+	(616.3578, 50059.41) (617.3611, 15758.59) (594.3764, 170028.23) (595.3793, 52255.38) (596.3819, 11953.29) (597.3852, 1868.63) (611.4076, 297.2)
C34 H55 N12 O3	−2.491	2.13E–04	−1.067	N.S.	C18+	(702.4314, 4643.8) (703.4354, 1840.41) (680.4592, 6949.51) (681.4605, 3260.07) (682.4631, 882.31)
C32 H51 O10 Esi + 12.411007	−2.027	1.73E–03	−1.121	N.S.	C18+	(618.3381, 4348.9) (619.3419, 1693.53) (596.3551, 18953.5) (597.3587, 6100.73) (598.3611, 1816.91) (599.3599, 222.98)
C31 H49 O9	−2.004	3.21E–03	−1.100	N.S.	C18+	(588.3267, 17685.77) (589.3303, 5836.98) (590.3389, 1836.92) (1153.6628, 1279.33) (1154.6656, 840.86) (566.3452, 135593.83) (567.3482, 40496.61) (568.3516, 8898.48) (569.3551, 1504.54) (1131.6823, 1156.18) (1132.6847, 661.48)
PE [22:6(4Z,7Z,10Z,13Z, 16Z,19Z)/0:0] Esi + 10.902	−8.687	4.61E–03	1.258	N.S.	C18+	(548.2743, 95660.68) (549.2773, 29194.23) (526.2926, 565743.44) (527.2959, 167649.31) (528.2982, 29241.65)
PE [22:6(4Z,7Z, 10Z,13Z,16Z,19Z)/0:0]	−8.597	4.81E–03	1.258	N.S.	C18+	(548.2743, 95660.68) (549.2773, 29194.23) (526.2926, 565743.44) (527.2959, 167649.31) (528.2982, 29241.65)
C20 H16 N O6	−2.765	9.37E–04	1.059	N.S.	HILIC−	(365.0906, 6087.18) (366.094, 981.33)
PC [18:3(6Z,9Z,12Z)/ 20:4(8Z,11Z,14Z,17Z)]	139.405	2.04E–02	−13.074	N.S.	HILIC+	(826.5427, 424.52) (804.552, 2422.12) (805.556, 1161.39)
PE [19:0/22:6(4Z,7Z, 10Z,13Z,16Z,19Z)]	2.840	1.86E–03	−1.052	N.S.	C18+	(828.5514, 124966.67) (829.5544, 61987.04) (830.5574, 16763.68) (831.5606, 3728.36)
Barbituric acid, 5-ethyl-5-(2-hydroxyethyl)-	2.247	1.87E–03	−1.127	N.S.	HILIC+	(223.069, 7991.25) (224.0709, 1855.2) (201.0871, 12020.45) (202.091, 1167.57)
PC [17:1(9Z)/ 20:5(5Z,8Z,11Z,14Z,17Z)]	2.245	3.26E–03	−1.191	N.S.	C18+	(814.5356, 84342.96) (815.5387, 42522.77) (816.542, 11650.13) (817.5466, 2508.01) (1606.0784, 4863.3) (1607.0813, 5175.72) (1608.085, 2997.93) (1609.0872, 1133.71) (1610.0887, 307.46) (792.5542, 853601.8) (793.5578, 419070.66) (794.5605, 109935.91) (795.5629, 21364.05) (796.5681, 3906.1) (797.5746, 744.92) (1584.097, 9308.74) (1585.1006, 9151.15) (1586.1033, 4983.11) (1587.1051, 2043.59) (1588.1064, 571.09)
PC [18:3(9Z,12Z,15Z)/22:4 (7Z,10Z,13Z,16Z)]	2.149	1.91E–03	−1.043	N.S.	C18+	(854.5667, 23313.19) (855.5701, 12648.29) (856.5735, 3862.51) (857.578, 946.06) (832.5849, 101901.32) (833.5881, 51366.33) (834.5911, 15361.98) (835.5943, 3344.88) (836.6016, 790.0)
Ancitabine Esi + 3.470998	543.089	1.69E–04	6.087	N.S.	HILIC+	(226.0825, 5276.52) (227.0869, 802.4)
Anibine	286.991	2.55E–03	2.066	N.S.	HILIC+	(226.045, 2249.78) (227.0482, 103.85) (429.0144, 412.48) (204.0632, 922.09)
C24 H19 N19 O4	110.749	5.77E–03	4.714	N.S.	HILIC−	(636.1795, 2567.15) (637.1818, 682.69)
C25 H13 N6 O3	76.653	1.47E–02	2.014	N.S.	HILIC−	(444.0975, 2311.36) (445.1024, 561.37)
PC [22:6(4Z,7Z,10Z,13Z,16Z,19Z)/ 22:6(4Z,7Z,10Z,13Z,16Z,19Z)]	2.530	4.69E–04	1.157	N.S.	C18+	(900.5547, 1268.98) (901.551, 921.35) (902.5487, 329.27) (878.5694, 15051.85) (879.5725, 8619.16) (880.5738, 3269.69) (881.5759, 1019.28)
PE [22:6(4Z,7Z,10Z,13Z,16Z,19Z)/ 22:4(7Z,10Z,13Z,16Z)]	2.206	5.36E–04	1.106	N.S.	C18+	(862.5356, 3496.06) (863.5376, 1840.5) (864.5534, 666.24) (840.5538, 14033.28) (841.5571, 7698.2) (842.5743, 3260.31)
PE [22:6(4Z,7Z,10Z,13Z,16Z,19Z)/ 22:6(4Z,7Z,10Z,13Z,16Z,19Z)]	2.503	6.34E–04	1.140	N.S.	C18+	(858.5046, 3908.22) (859.5076, 2355.39) (860.5147, 904.48) (861.513, 153.03) (836.5229, 15859.57) (837.5261, 8592.62) (838.5362, 4116.88) (839.5368, 919.5)
Quercetin 3,4′-dimethyl ether 7-glucoside	2.100	2.90E–03	1.303	N.S.	HILIC−	(491.1188, 9107.03) (492.1217, 2024.41)
C6 H10 N2 Esi + 3.0780017	−1.620	N.S.	−2.267	7.90E–03	C18+	(111.0915, 9416.54) (112.0944, 670.02)
C33 H39 N14 O	−2.807	N.S.	−1.585	1.09E–02	C18+	(670.3348, 81.0) (648.3504, 17316.61) (649.3547, 6659.75) (665.3842, 919.13)
C15 H39 N16	28.361	N.S.	598.849	2.73E–04	HILIC+	(444.3576, 4252.18) (445.3726, 746.13) (446.3756, 96.88)
C18 H39 N9	−1.073	N.S.	3.925	9.48E–04	HILIC+	(785.6224, 602.19) (382.3266, 5576.34) (383.3276, 2716.83) (399.3647, 8642.28) (400.3722, 2169.87) (780.6662, 3471.65) (781.6672, 1776.56) (764.6397, 5252.95)
Docusate	1.553	N.S.	3.088	1.69E–04	HILIC−	(421.2257, 12808.72) (422.2282, 3206.75) (423.2247, 955.26)

**Metabolic signatures with raw p-value ≤ 0.05 and fold change (FC) > 2.0 or <−2.0 induced by LPS administration in control and/or Abca7^±^ mice are listed (N = 6/each). N.S., not significant*.*

### Identification of Metabolite Modules Associated With ABCA7 Haplodeficiency and LPS Stimulation in Mouse Brains

**FIGURE 2:**
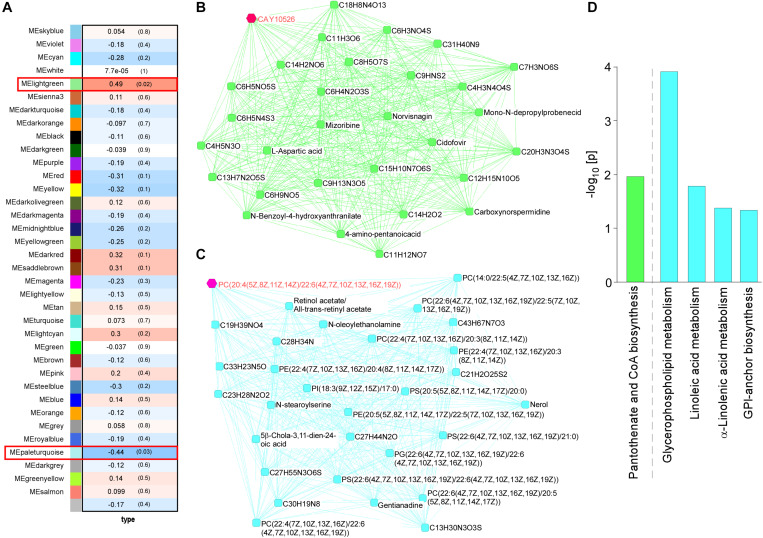
Impact of ABCA7 haplodeficiency and peripheral LPS stimulation on mouse cortical metabolomes. **(A)** The correlation between metabolite module eigengenes and the four groups of mice: (1) control with LPS administration, (2) *Abca7*^±^ with LPS administration, (3) control with LPS administration, and (4) *Abca7*^±^ with LPS administration. Each module is represented with a unique color. The module traits were correlated with the four groups of mice (1–4). The corresponding correlations and *P*-values are displayed in each module. **(B,C)** The interactions of top 30 hub metabolites within the lightgreen **(B)** and paleturquoise **(C)** modules were visualized. **(D)** Pathway analysis of metabolites using the KEGG database. Pathways in the lightgreen **(B)** and paleturquoise **(C)** modules are shown (raw *p*-value < 0.05).

WGCNA for the brain metabolites identified two modules that were changed among four mouse groups (control and *Abca7*^±^ mice with or without LPS administration): lightgreen (*p* = 0.02) and paleturquoise (*p* = 0.03) (Figure 2A). In the analysis, the lightgreen module contains CAY10526, C6 H3 N O4 S, C9 H N S2, C4 H3 N4 O4 S, and C6 H5 N4 S3 as the top 5 ranked hub metabolites (Figure 2B), although most of them are classified as unknown metabolites. On the other hand, the paleturquoise module was relatively enriched with metabolites related to lipid metabolism, which include PC[20:4(5Z,8Z,11Z,14Z)/22:6(4Z,7Z, 10Z,13Z,16Z,19Z)], Retinol acetate/All-*trans*-retinyl acetate, PC[22:6(4Z,7Z,10Z,13Z,16Z,19Z)/ 22:5(7Z,10Z,13Z,16Z,19Z)], C28 H34 N, and C13 H30 N3 O3 S as the top 5 ranked hub metabolites (Figure 2C). The KEGG pathway analysis demonstrated that “Glycerophospholipid metabolism” was enriched by metabolites in the paleturquoise module (FDR = 1.02E–02). A few other enriched pathways include “Linoleic acid metabolism,” “α-Linolenic acid metabolism,” and “Glycosylphosphatidylinositol (GPI)-anchor biosynthesis” with nominal significance (raw *p* < 0.05). Metabolites in the lightgreen module are suggested to be associated with “Pantothenate and CoA biosynthesis” with nominal significance (raw *p* < 0.05) (Figure 2D). Since glycerophospholipids and GPI are major components of the cell membrane, these results suggest that ABCA7 is involved in the pathways related to polyunsaturated fatty acid conversion from membrane phospholipids during LPS-induced inflammation.

### ABCA7 Haplodeficiency Modulates the Fatty Acid Metabolism in the Brain During LPS-Induced Inflammation

**FIGURE 3:**
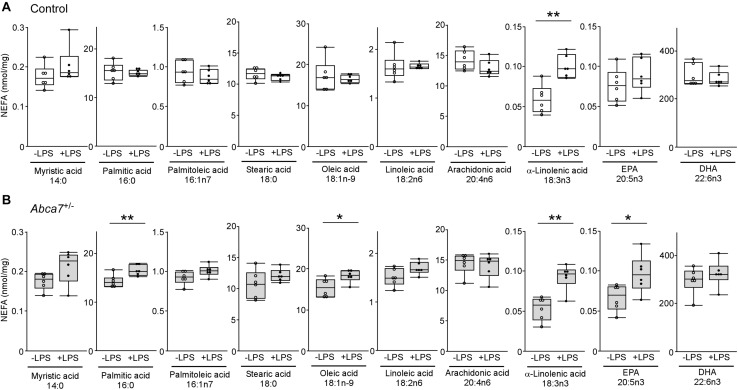
Altered effects of LPS stimulation on brain non-esterified fatty acid levels in heterozygous ABCA7 knockout mice. The amounts of non-esterified fatty acids (NEFAs) were analyzed through mass spectrometry in the cortex of control (**A**; white) and *Abca7*^±^ (**B**; gray) mice administrated with or without LPS. Concentration of myristic acid, palmitic acid, palmitoleic acid, stearic acid, oleic acid, linoleic acid, arachidonic acid, α-linolenic acid, eicosapentaenoic acid (EPA), and docosahexaenoic acid (DHA) were plotted. All NEFA concentrations were normalized to the protein levels. Horizontal lines, boxes, and whiskers correspond to median, interquartile range (IQR), and the furthest points within 1.5 × IQR from the box, respectively (*N* = 6/each). *p < 0.05, **p < 0.01, by student-*t* test.

To further investigate how ABCA7 haplodeficiency influences the LPS-induced metabolism of polyunsaturated fatty acids in the brain, amounts of non-esterified fatty acid were measured by mass spectrometry in the cortex of control (Figure 3A) and *Abca7*^±^ (Figure 3B) mice with or without the LPS administration. Among the 12 major analytes, 10 non-esterified fatty acids were detectable in the mouse cortical samples, which include myristic acid 14:0, palmitic acid 16:0, palmitoleic acid 16:1n7, stearic acid 18:0, oleic acid 18:1n-9, linoleic acid 18:2n6, α-linolenic acid 18:3n3, arachidonic acid 20:4n6, eicosapentaenoic acid (EPA) 20:5n3 and docosahexaenoic acid (DHA) 22:6n3, but not palmitelaidic acid and elaidic acid. The amount of α-linolenic acid increased upon LPS stimulation in the cortex of control mice, although there were no significant effects on other fatty acids (Figure 3A). In contrast, we found that LPS administration induced the upregulations of EPA, oleic acid and palmitic acid in addition to α-linolenic acid in the cortex of *Abca7*^±^ mice (Figure 3B). These results indicate that ABCA7 plays a critical role in the brain metabolism of EPA, oleic acid and palmitic acid during LPS-induced acute inflammation.

## Discussion

ATP-binding cassette transporters play a critical role in the metabolism of inflammatory lipid mediators including leukotrienes and prostaglandins (Van De Ven et al., 2009). Indeed, our results revealed that ABCA7 haplodeficiency modulates metabolism of EPA, oleic acid and palmitic acid in the brain upon LPS stimulation. EPA, α-linolenic acid and DHA are major omega-3 fatty acids, which have strong biological functions in diverse pathways. Omega-3 and omega-6 polyunsaturated fatty acids are taken from various foods in the form of triglycerides and integrated into cellular lipids such as triacylglycerol, cholesterol esters and phospholipids (Kaur et al., 2014). Of note, those polyunsaturated fatty acids are abundantly localized in major immune cells including macrophages, neutrophils and lymphocytes (Gutierrez et al., 2019). Although proinflammatory eicosanoids are produced from omega-6 arachidonic acid in the acute phase of inflammation, omega-3 EPA and DHA mediates anti-inflammatory functions and are involved in the resolution phase of inflammation (Serhan, 2014; Astudillo et al., 2019). We found that brain EPA levels were upregulated in *Abca7*^±^ mice 3.5 h after the LPS injection, whereas there was no significant increase observed in control mice. Thus, the compromised acute immune responses observed in the brain of *Abca7*^±^ mice (Aikawa et al., 2019) may be partially due to the increase of EPA as a pro-resolving lipid mediator. There was no evident difference in the baseline levels of brain EPA between control and *Abca7*^±^ mice, but α-linolenic acid levels were increased by LPS stimulation in both groups. Thus, ABCA7 haplodeficiency may increase EPA production during acute inflammation by accelerating the interaction of phospholipids containing EPA with phospholipase A2 on the cell membrane, although further studies are needed for clarification.

In addition, increases of oleic acid and palmitic acid were also observed in the brains of *Abca7*^±^ mice following LPS stimulation, but not in control mice. Given that phospholipids incorporated with oleic acid or palmitic acid are major components of the cell membrane (Lopez et al., 2014), ABCA7 may be involved in the pathways for the metabolism of those fatty acids in activated immune cells. Whereas oleic acid has likely both proinflammatory and anti-inflammatory effects depending on the cell type, it inhibits LPS-induced NF-κB transactivation in microglial cells (Oh et al., 2009). Thus, the increase of brain oleic acid is consistent with the observations of immune suppressive phenotypes of *Abca7*^±^ mice. In contrast, palmitic acid has been shown to rather enhance the inflammatory responses and induce endoplasmic reticulum (ER) stress (Korbecki and Bajdak-Rusinek, 2019). The effect of palmitic acid on LPS-induced acute neuroinflammation may be compromised by other factors. Nonetheless, we previously found that aged *Abca7*^–/–^ mice have higher brain levels of PERK and phosphorylated eIF2α compared to control mice (Sakae et al., 2016). Thus, ABCA7 deficiency may alter brain palmitic acid metabolism, which is involved in exacerbated ER stress under pathological conditions.

In summary, our results demonstrated that ABCA7 haplodeficiency substantially influences the metabolomics profile of mouse brains after LPS stimulation. Since fatty acids with anti-inflammatory effects including EPA and oleic acid were specifically increased in *Abca7*^±^ mice upon LPS stimulation, ABCA7 haplodeficiency possibly facilitates the shift from pro-inflammatory phase to resolution phase during acute inflammation. Further studies should refine how ABCA7 deficiency influences the brain metabolism of specialized pro-resolving mediators at different time points after LPS stimulation. When activations of the immune system are excessively repeated and/or prolonged, it is possible that ABCA7 loss of function exacerbates the depletion of resources for anti-inflammatory fatty acids, thereby increasing the risk of AD due to insufficient immune resolutions. Indeed, levels of EPA and oleic acids have been noted to be lower in the inferior temporal gyri of AD cases compared to cognitively unimpaired subjects as early as at the asymptomatic stage (Snowden et al., 2017). Prospective epidemiological studies also show the strong causal association between low consumption of fish and/or low DHA intake and AD (Avallone et al., 2019). Therefore, determining the molecular mechanisms for which ABCA7 loss of function causes the dysregulation of fatty acid metabolism should provide important clues to understand the pathogenesis of AD and to develop novel therapeutic strategies for combatting the disease. Although some clinical trials involving the administration of omega-3 fatty acids (DHA + EPA) fail to show therapeutic effects in treating AD (Avallone et al., 2019), early interventions through nutritional approaches may be a potential strategy to prevent the development and progression of symptoms in AD patients, particularly those carrying *ABCA7* loss of function variants.

## Data Availability Statement

The original contributions generated for this study are included in the article/Supplementary Materials, further inquiries can be directed to the corresponding author.

## Ethics Statement

The animal study was reviewed and approved by Mayo Clinic IACUC.

## Author Contributions

TA and TK designed the research studies. TA, YR, and M-LH conducted experiments and acquired data. TA, YR, YA, AA, MF, GB, and TK analyzed the data. TA and TK wrote the first draft. All authors contributed to writing the final manuscript.

## Conflict of Interest

The authors declare that the research was conducted in the absence of any commercial or financial relationships that could be construed as a potential conflict of interest.
